# MLVI-CNN: a hyperspectral stress detection framework using machine learning-optimized indices and deep learning for precision agriculture

**DOI:** 10.3389/fpls.2025.1631928

**Published:** 2025-09-17

**Authors:** Poornima S, A. Shirly Edward

**Affiliations:** Department of Electronics Communication Engineering, SRM Institute of Science and Technology, Vadapalani Campus, Chennai, India

**Keywords:** hyperspectral imaging, vegetation index, machine learning, crop stress, early detection, remote sensing

## Abstract

**Introduction:**

Early and accurate detection of crop stress is vital for sustainable agriculture and food security. Traditional vegetation indices such as NDVI and NDWI often fail to detect early-stage water and structural stress due to their limited spectral sensitivity.

**Method:**

This study introduces two novel hyperspectral indices — Machine Learning-Based Vegetation Index (MLVI) and Hyperspectral Vegetation Stress Index (H_VSI) — which leverage critical spectral bands in the Near-Infrared (NIR), Shortwave Infrared 1 (SWIR1), and Shortwave Infrared 2 (SWIR2) regions. These indices are optimized using Recursive Feature Elimination (RFE) and serve as inputs to a Convolutional Neural Network (CNN) model for stress classification.

**Results:**

The proposed CNN model achieved a classification accuracy of 83.40%, effectively distinguishing six levels of crop stress severity. Compared to conventional indices, MLVI and H_VSI enable detection of stress 10–15 days earlier and exhibit a strong correlation with ground-truth stress markers (r = 0.98).

**Discussion:**

This framework is suitable for deployment with UAVs, satellite platforms, and precision agriculture systems.

## 1 Introduction

Sustainable crop production is increasingly challenged by abiotic stresses such as drought, nutrient deficiency, and heat. These stresses often lead to significant yield losses if not detected and mitigated early (Koh et al., 2022). Traditional monitoring techniques, including normalized difference vegetation index (NDVI) and normalized difference water index (NDWI), focus primarily on chlorophyll content and have limited capability in detecting early-stage or non-chlorophyll-related stress responses (Sun et al., 2017; Lu et al., 2020). The authors (Varghese et al., 2021) reviewed the capabilities of Sentinel-2 for drought detection, reinforcing the need to advance beyond traditional multispectral sensors toward hyperspectral solutions for early stress detection (Pertiwi et al., 2024). Hence, there is a strong need for more sensitive and accurate detection tools.

Hyperspectral imaging (HSI) has emerged as a transformative technology in remote sensing, offering high spectral resolution that can detect subtle physiological changes in crops. With hundreds of contiguous spectral bands, HSI captures detailed reflectance patterns sensitive to plant water status, canopy structure (Lutz et al., 2024), and stress-related biochemical properties (Okyere et al., 2024). These spectral signatures, especially in the Near-Infrared (NIR) and Shortwave Infrared (SWIR) regions, are particularly useful for monitoring crop stress (You et al., 2024).

Integrating machine learning (ML) and deep learning (DL) techniques with HSI has further enhanced its potential, enabling automated feature selection and robust classification performance (Raja et al., 2022). For instance, convolutional neural networks (CNNs) and support vector machines (SVMs) have been widely used to analyze spectral-spatial information for early disease and stress detection in crops (Varghese et al., 2021; Hui et al., 2023).

Despite these advancements, most existing approaches lack generalizability across different stress types and fail to provide early warnings. Moreover, the indiscriminate use of all spectral bands increases computational overhead and reduces model interpretability (Wei et al., 2021). Studies have shown that redundant spectral information can negatively impact classifier performance unless optimal band selection is applied (Benelli et al., 2020).

To bridge these gaps, we propose two novel hyperspectral vegetation indices—Machine Learning-Based Vegetation Index (MLVI) and Hyperspectral Vegetation Stress Index (H_VSI)—that leverage recursive feature elimination (RFE) for data-driven band selection. These indices, when fed into a 1D CNN classifier, enable efficient and accurate early stress detection. The proposed CNN model achieved a classification accuracy of 83.40% and successfully differentiated six levels of crop stress severity.

This study aims to:

- Develop machine learning-optimized vegetation indices using RFE;
- Integrate these indices into a 1D CNN model for multi-class stress classification; and
- Evaluate the proposed framework against conventional indices using hyperspectral data.

The rest of the paper is structured as follows: Section 2 discusses the related works; Section 3 outlines the methodology, including data acquisition, preprocessing, and feature selection; Section 4 presents the results and discussion, including index performance, classification metrics, and geospatial stress mapping; Section 5 concludes the study and discusses future directions for precision agriculture deployment.

## 2 Related work

### 2.1 Hyperspectral imaging in crop stress detection

Hyperspectral imaging (HSI) captures reflectance across hundreds of narrow bands in the visible, near-infrared (NIR), and short-wave infrared (SWIR) regions, enabling detection of early stress-induced changes in plant physiology. Multiple studies have shown that stress-related alterations—such as reductions in leaf water content, pigment degradation, and changes in canopy structure—correlate with spectral variations, particularly in the SWIR region (Koh et al., 2022). The author (Okyere et al., 2024) demonstrated that hyperspectral imaging combined with ML models effectively captured both drought and nitrogen stress interactions in wheat, highlighting its dual diagnostic potential. Studies like You et al. (2024) emphasize the role of surface parameterization and its influence on remote sensing outputs, which can be crucial when interpreting hyperspectral data in variable terrain or microclimates. UAV-mounted HSI systems offer fine spatial resolution and large-area coverage, making them ideal for early stress detection across a range of crops, including wheat, pearl millet, potato, and maize (Khanna et al., 2019). The work (Wei et al., 2021) showed how point-based hyperspectral readings, when combined with multivariate regression, could effectively estimate grapevine water status, underscoring the value of spectral resolution for water stress analysis.

### 2.2 Machine and deep learning for stress classification

Machine learning (ML) models like Support Vector Machines (SVM), Random Forest (RF), and Deep Neural Networks (DNN) have been successfully used for classifying crop stress using HIS (Lu et al., 2020). For instance, SVMs have achieved high accuracy (>90%) in detecting drought and nutrient deficiencies (Varghese et al., 2021). However, their performance relies heavily on the quality and selection of input features.

Deep learning models, especially 1D and 2D Convolutional Neural Networks (CNNs), have demonstrated strong potential in extracting hierarchical features directly from raw or preprocessed spectral data. CNNs outperform traditional ML in cases with large datasets but require considerable computational power and risk overfitting when spectral inputs are not optimized (Wei et al., 2021). AutoML and meta-learning approaches have also shown promise in learning from limited training data (Benelli et al., 2020).

Recent advancements in ensemble learning and hybrid deep models have demonstrated the power of integrating multiple learning paradigms for complex classification tasks (Chen B. et al., 2024). For example (Bao et al., 2023), proposed a Deep Forest (DF)-based model for Golgi protein classification, which combines decision tree ensembles with representation learning, offering robustness with limited data and avoiding the need for extensive hyperparameter tuning—an approach that is well-suited for hyperspectral stress analysis where labeled data is limited (Chen H. et al., 2024). Similarly (Duarte-Carvajalino et al., 2021), utilized feature-engineered inputs combined with machine learning classifiers for cleft lip and palate reconstruction, demonstrating the value of domain-specific feature extraction in improving model interpretability and classification accuracy. These insights align with our study’s design, where Recursive Feature Elimination (RFE) is used to derive optimized hyperspectral vegetation indices (MLVI, H_VSI) that feed into a CNN for robust stress classification.

### 2.3 Vegetation index development

Traditional vegetation indices such as NDVI and NDWI are limited by their reliance on broad spectral bands and chlorophyll sensitivity, which hampers early detection of abiotic stress. Recent research has focused on developing more stress-specific indices by targeting NIR and SWIR wavelengths linked to water content, leaf thickness, and pigment loss (Murphy et al., 2020). Examples include the Leaf Water Vegetation Index (LWVI), which is designed for drought monitoring in durum wheat, and indices optimized for pearl millet and groundnut using ML-based feature selection (Kim et al., 2015; Malounas et al., 2024).

Despite these efforts, most indices remain manually crafted and are not dynamically optimized for varying crop conditions or stress types. Moreover, few studies incorporate both band selection and index formulation into a unified classification framework.

### 2.4 Crop-specific applications

In wheat, hyperspectral indices combined with SVM or RF classifiers have classified drought stress levels under varying nitrogen regimes with accuracies exceeding 94% (Roy et al., 2023). In maize, unsupervised learning techniques have detected stress up to 10 days earlier than NDVI (Gessner et al., 2023). In potato, CNN and RF models trained on hyperspectral features have predicted water stress with high precision, even on small datasets. In tomato crops, author (Zhang et al., 2024; Tola et al., 2025) used spectral indices to assess salinity stress, demonstrating the broader utility of reflectance-based stress diagnosis across crop types.

These findings highlight the cross-crop applicability of HSI and ML models but also point to a need for better generalization and computational efficiency.

### 2.5 Research gap and study contribution

While existing methods demonstrate strong performance, they often rely on fixed-band inputs and perform binary classification of stress presence. They lack the ability to generalize across diverse stress types or quantify stress severity (Zhou et al., 2022). Moreover, full-spectrum HSI models introduce unnecessary computational complexity and limit scalability in real-world deployments.

This study bridges these gaps by:

Proposing two new hyperspectral vegetation indices (MLVI and H_VSI) derived from machine learning-guided Recursive Feature Elimination (RFE); Embedding these indices into a CNN classifier for accurate, multi-level stress classification; Validating the model on real-world UAV-acquired hyperspectral datasets, demonstrating its practical value in precision agriculture (Abbas et al., 2023).

This **Table 1** highlights the diverse applications of machine learning techniques in hyperspectral-based crop stress detection, demonstrating their effectiveness across various crops and stress conditions.

**Table 1:** Comparison of machine learning techniques for hyperspectral stress detection.

Machine Learning Technique	Application in Crop Stress Detection	Citation
Support Vector Machines (SVM)	Classification of drought stress levels in wheat and pearl millet	(Sankararao et al., 2023)
Random Forest (RF), Deep Neural Networks (DNN)	Prediction of stomatal conductance and photosynthetic rates in wheat	(Okyere et al., 2024)
Convolutional Neural Networks (CNN)	Identification of water stress in Chickpea	(Sankararao et al., 2021)
AutoML	Classification of broccoli drought acclimation/stress responses	(Malounas et al., 2024)
Meta-Learning	Detection of frost stress in tomato plants with limited target domain data	(Ruan et al., 2023)

Traditional vegetation indices, such as NDVI and NDWI, have been widely used for vegetation monitoring. However, their effectiveness in detecting early stress conditions is limited due to their reliance on broad spectral bands (Das et al., 2023). Recent advancements in hyperspectral imaging and machine learning have enabled more precise stress detection by leveraging narrow spectral features. Studies have demonstrated that hyperspectral indices incorporating SWIR bands can detect stress earlier than NDVI. Machine learning-based approaches, such as Random Forest, CNN have further improved classification accuracy by automatically selecting the most relevant spectral bands.

## 3 Methodology

**Figure 1:**
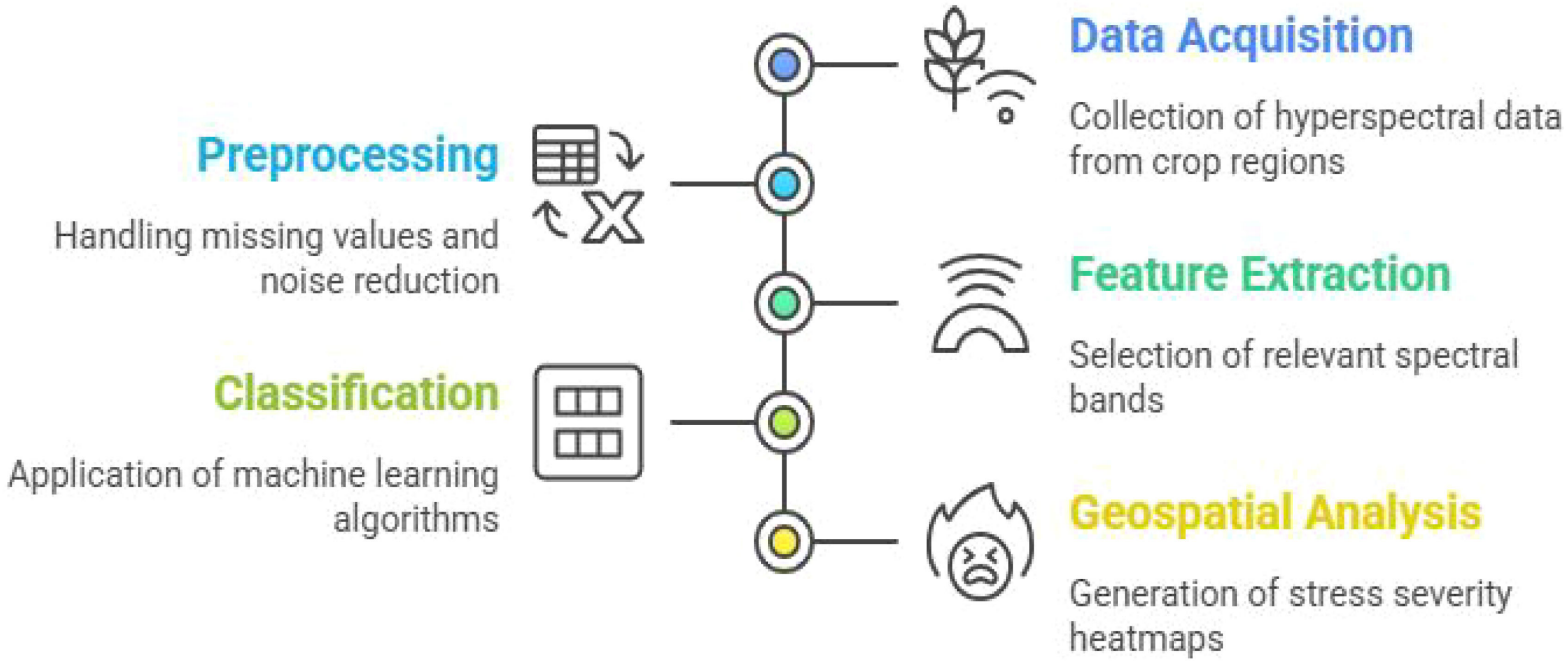
General workflow of crop stress analysis.

**Figure 2:**
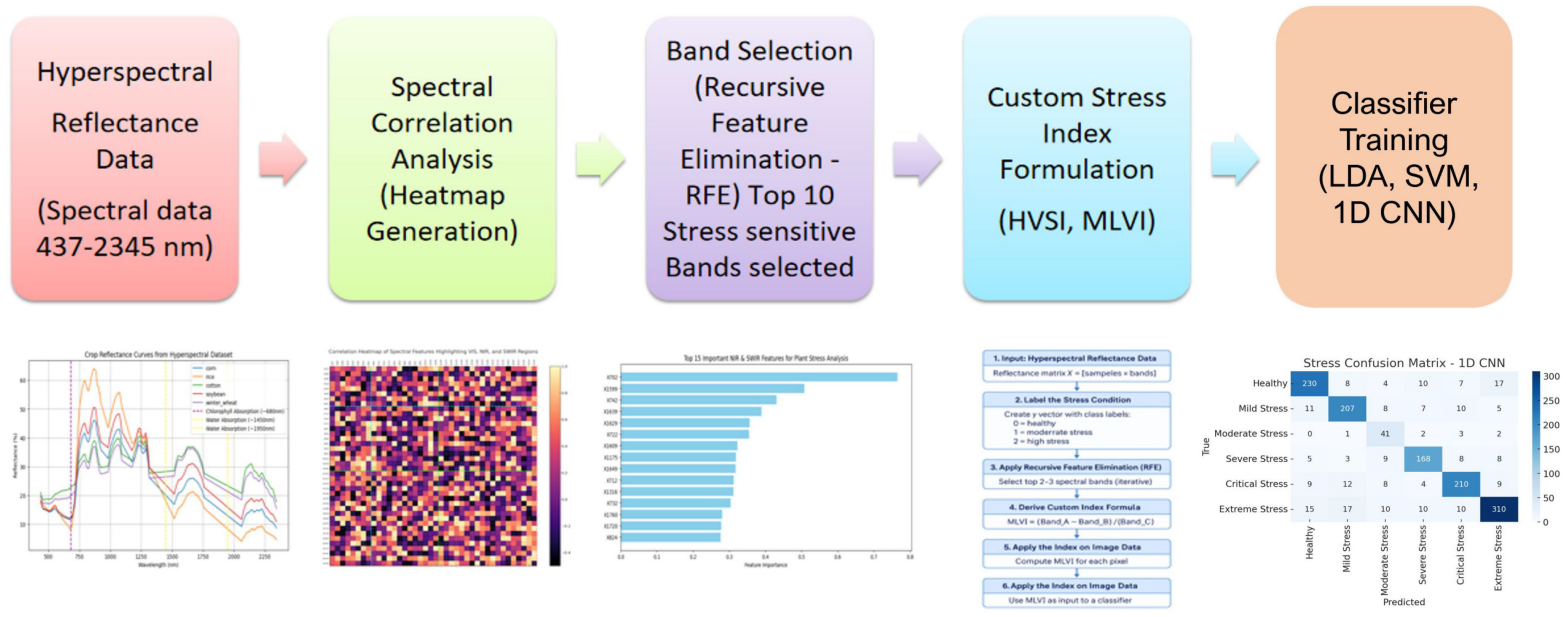
Workflow diagram of the proposed methodology for early crop stress detection.

This section outlines the complete workflow for hyperspectral stress classification, including data acquisition details, preprocessing, feature selection, vegetation index formulation, and deep learning-based classification as mentioned in **Figures 1**, **2**.

### 3.1 Dataset description

This study utilizes the GHISACONUS Hyperspectral Spectral Library, comprising reflectance data acquired from NASA’s EO-1 Hyperion sensor. The dataset spans a spectral range of 437–2345 nm and includes detailed metadata on location, crop type, and growth stage. Key bands relevant to plant stress were identified, including:

X661 (Red) – chlorophyll absorption,
X854 (NIR) – canopy structure,
X1649 (SWIR1) – water content,
X2133 (SWIR2) – leaf dryness.

### 3.2 Spectral preprocessing

To enhance signal quality, spectral reflectance data were first smoothed using the Savitzky-Golay filter (Sara and Rajasekaran, 2025), which fits a low-degree polynomial across a moving window to reduce noise while preserving peak shapes. This step ensures that small but meaningful spectral variations related to stress are retained. Following smoothing, Z-score normalization was applied to each spectral band (Alordzinu et al., 2021; Johnson et al., 2024b). This standardized the input by centering values around a mean of zero and scaling them to unit variance, which is essential for preventing bias during model training.

#### 3.2.1 Savitzky-Golay filtering

In this study, the Savitzky-Golay filter was applied during the preprocessing stage to enhance the quality of the hyperspectral reflectance data. This step was essential for minimizing noise introduced by environmental variability, sensor drift, and atmospheric interference, which can distort subtle spectral cues relevant to early stress detection. By smoothing the data while retaining key spectral patterns, the filter improved the reliability of subsequent feature selection and classification stages.

The polynomial smoothing equation (Equation 1) enhances the reflectance signal by:

**Savitzky-Golay formula:**


(1) $$\hat{y}i=\sum \;j=-m^{m}c_{j}y_{i+j}$$

Where $\hat{y}i$ is the smoothed value, $y_{i+j}$ are neighboring points, and $c_{j}$ are the filter coefficients.

This would enhance the scientific rigor of the preprocessing explanation.

**Figure 3:**
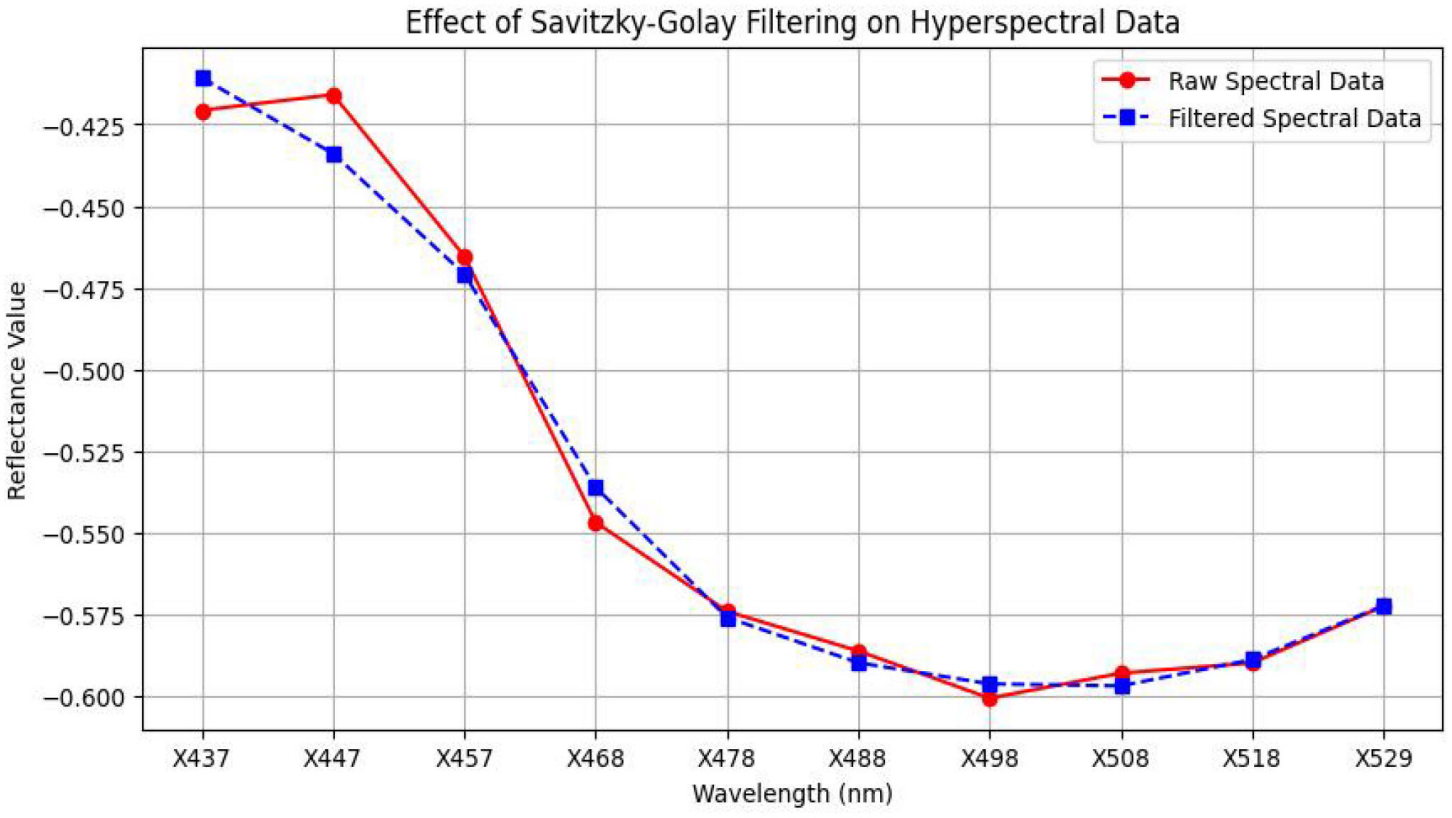
Effect of savitzky-golay filtering on hyperpsectral data.

**Figure 3** demonstrates the effect of the filter:

- The raw spectral data (red line with circles) shows noticeable fluctuations and noise.
- The smoothed spectral curve (blue dashed line with squares) closely follows the overall trend of the raw data but with significantly reduced variability.
- This preprocessing step ensured that the spectral input to machine learning and vegetation index calculations remained biologically meaningful and robust to noise.

Then apply Z-score normalization to ensure that the filtered spectral data is scaled consistently across bands before feature selection and CNN input.

#### 3.2.2 Normalization: scaling of reflectance values across spectral bands

To ensure consistency and stability across the high-dimensional hyperspectral inputs, Z-score normalization was applied to all selected spectral bands prior to index formulation and CNN classification. This standardization technique transforms each spectral value by subtracting the mean and dividing by the standard deviation of its respective band. As hyperspectral reflectance data often contains varying magnitudes across wavelengths due to differences in sensor response and biophysical properties, normalization is essential for preventing feature dominance and improving model convergence.

This preprocessing step plays a critical role in our pipeline, especially when feeding selected bands (e.g., X854, X1649, X2133) or derived indices (MLVI and H_VSI) into the 1D CNN. By centering the data around zero mean and scaling to unit variance, the model is able to learn more efficiently from all spectral features without bias toward specific band ranges.

**Z-score normalization was applied using** **Equation 2****:**


(2) $$X_{norm}=\frac{X-\mu }{\sigma }$$

Where:

X = original reflectance value
μ = mean of the spectral band
σ = standard deviation of the spectral band

### 3.3 Spectral correlation analysis

A spectral correlation matrix was generated to visualize redundancy and inter-band relationships. Bands exhibiting strong correlation with known stress indicators were prioritized for further analysis. Heatmaps were used to identify regions with high information density and eliminate irrelevant wavelengths.

**Figure 4:**
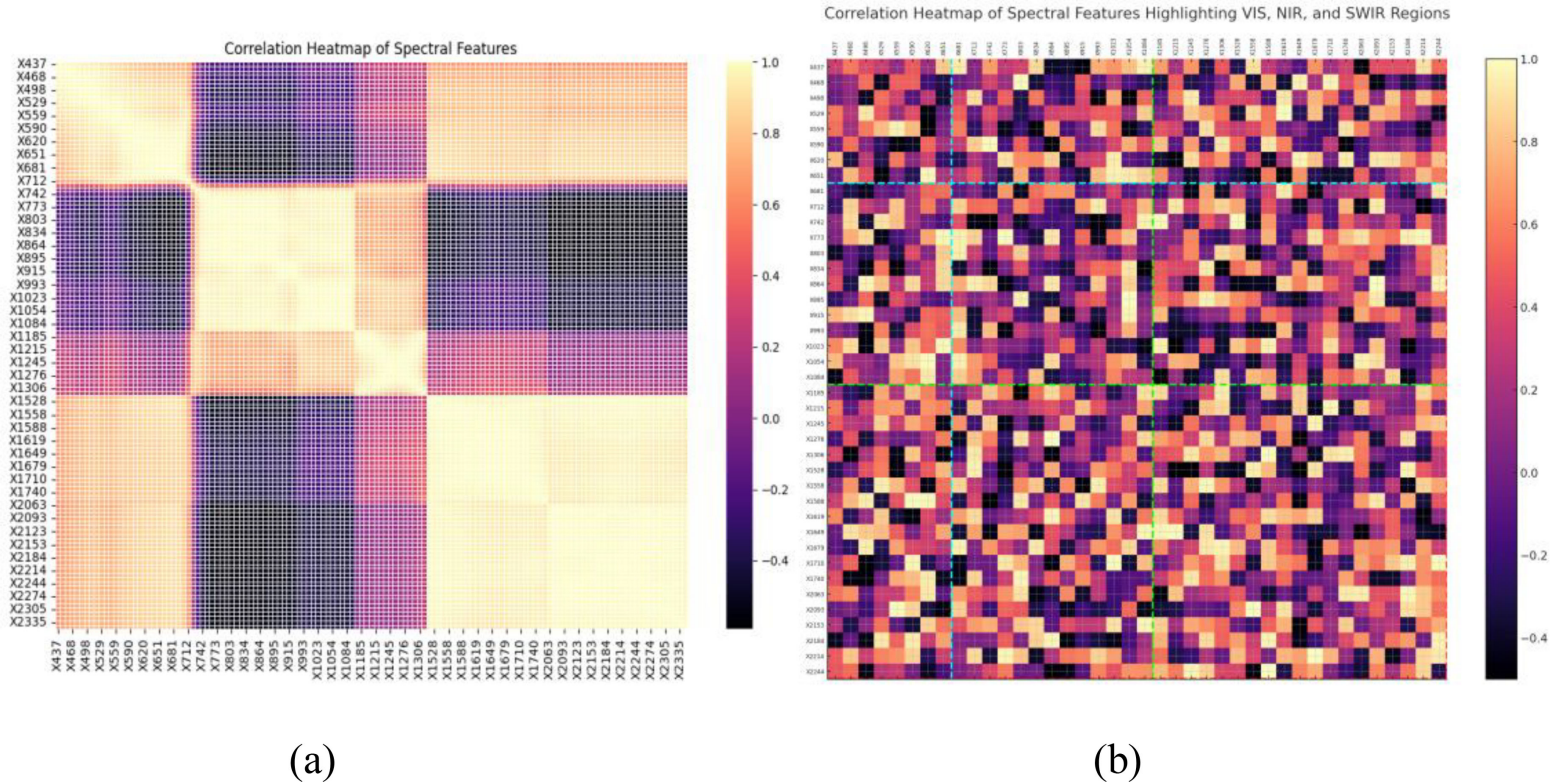
**(a)** Correlation heatmap of hyperspectral features in the dataset **(b)** Features highlighting VIS and SWIR Bands.

Correlation heatmap of hyperspectral features with regions corresponding to visible-range (VIS), near-infrared (NIR), and short wave IR (SWIR) bands highlighted (**Figure 4a**). Strong intra-region correlations are observed within VIS and NIR bands, while SWIR bands show distinct correlation behavior (**Figure 4b**), indicating their importance for water and structural stress detection (Kumar and Shankar, 2024). The highlighted divisions validate the selection of multi-spectral bands for optimal stress-sensitive index formulation.

The Pearson correlation coefficient r, computed using Equation (X), quantifies linear dependency between spectral bands. The correlation matrix (**Figure 4**) uses this metric to identify highly correlated band pairs for elimination, and bands with low redundancy and high stress sensitivity for retention.

Equation 3 or method used to compute correlation coefficients (Pearson or Spearman) is

**Pearson’s correlation coefficient formula:**


(3) $$\begin{array}{c} r=\frac{\sum_{i=1}^{n}(X_{i}-\bar{X})\left(Y_{i}-\bar{Y}\right)}{\sqrt{\sum_{i=1}^{n}{\left(X_{i}-\bar{X}\right)}^{2}}\cdot \sqrt{\sum_{i=1}^{n}{\left(Y_{i}-\bar{Y}\right)}^{2}}} \end{array}$$

Where,

$X_{i}$ and $Y_{i}$ are the individual sample points

$\bar{X}$ is the mean of the X values

$\bar{Y}$ is the mean of the Y values
n is the number of paired observations

**Figure 5:**
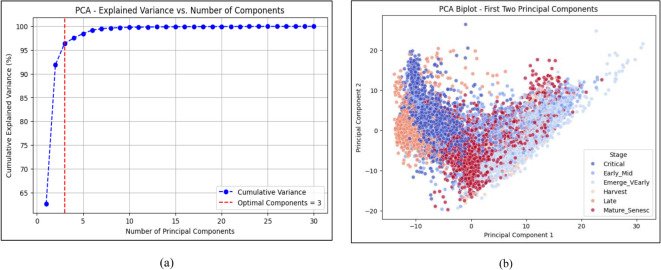
**(a)** PCA analysis and **(b)** PCA biplot.

Hyperspectral data has hundreds of bands, many of which are redundant or noisy. PCA reduces this to the top 30 components (Ruan et al., 2023), capturing the most important variance in the data. These components are used as input for training the CNN model. The plot (**Figures 5a, b**) shows how much total variance is retained as more principal components are added. First 3 components explain over 80% of the data variance. 30 components retain over 95%, ensuring minimal information loss.

The different vegetation indices and their respective sensitivities are summarized in **Table 2**. NDVI primarily reflects chlorophyll concentration but often exhibits delayed responses under stress conditions. NDWI enhances sensitivity to water stress, while H_VSI extends detection to early water and structural stress. The MLVI index, optimized through machine learning-driven band selection, offers superior early-stage stress detection by integrating multiple stress-sensitive spectral regions (Felix et al., 2025).

**Table 2:** Novel index comparison with traditional indices.

Index	Formula	Sensitivity
NDVI	(NIR - Red)/(NIR + Red)	Chlorophyll-based, delayed stress response
NDWI	(NIR - SWIR1)/(NIR + SWIR1)	Water stress detection
H_VSI	(NIR - SWIR1)/ (NIR + SWIR1 + SWIR2)	Early water & structural stress detection
MLVI	${\text{MLVI}}_{\text{gen}}=\frac{R_{\lambda_{1}}-R_{\lambda_{2}}}{R_{\lambda_{3}}}$	Machine-learning optimized stress detection

### 3.4 Band selection using recursive feature elimination

Recursive Feature Elimination (RFE) was used in conjunction with a Random Forest estimator to select the top 10 stress-sensitive spectral bands. This dimensionality reduction step improves both classification performance and model interpretability by focusing on biologically relevant features. The selected bands (e.g., X854, X1649, X2133) formed the foundation for vegetation index design.

Given the high dimensionality of hyperspectral data, feature reduction is essential to improve model performance and interpretability. Two complementary approaches were used:

- Recursive Feature Elimination (RFE): This method ranks spectral bands based on their importance in a machine learning model (e.g., SVM) and iteratively eliminates the least significant bands. The selected bands were used to construct two custom indices: MLVI and H_VSI. RFE band selection is explained in **Algorithm 1**.
- Principal Component Analysis (PCA): PCA was applied to visualize data variance and support unsupervised dimensionality reduction. While PCA outputs were not used directly in model training, they were used for exploratory analysis and variance validation (Lin et al., 2014).

Algorithm 1 **Stress-Sensitive band selection using recursive feature elimination (RFE).**


Input: Hyperspectral dataset X with spectral bands, Labels Y, Model M (e.g., Random Forest or SVM), Desired number of bands k
Output: Top k stress-sensitive spectral bands
1: Initialize feature set F ← all spectral bands in X
2: while |F| > k do
3:     Train model M using features in F
4:      Compute importance scores for each spectral band in F
5:       Remove the band with the lowest importance score from F
6: end while
7: SelectedBands ← F
8: Use SelectedBands to compute MLVI and H_VSI indices
9: Return SelectedBands

The hyperspectral data was first standardized and then reduced using Principal Component Analysis (PCA) to retain only the most informative features for CNN classification illustrates in **Figure 5**.

PCA selects the most important features from the hyperspectral dataset and gives the optimal feature extraction. After PCA selection the Band selection is carried over. For the better band selection Spectral Correlation Analysis is done for the dataset.

PCA transforms input data X into uncorrelated components using Equation 4:


(4) Z=X.W

Where:

X: standardized spectral data (after scaling)
W: matrix of eigen-vectors (principal components)
Z: transformed data (principal components)

### 3.5 Vegetation index formulation

Based on the RFE-selected bands, two novel vegetation indices were formulated:

- Machine Learning-Based Vegetation Index (MLVI): Combines bands most predictive of early stress from NIR and SWIR regions.
- Hyperspectral Vegetation Stress Index (H_VSI): Captures stress-sensitive spectral contrast using a weighted band-ratio approach optimized for drought and nutrient deficiencies.

**Hyperspectral Vegetation Stress Index (H_VSI), defined in** Equation 5:


(5) $$HVSI=\frac{\left(NIR-SWIR1\right)}{\left(NIR+SWIR1+SWIR2\right)}$$

This index effectively integrates NIR, SWIR1, and SWIR2 to detect stress levels, including water stress and structural damage. NDVI - based indices focus on chlorophyll (Red/NIR) but are weak for detecting water stress. H_VSI directly accounts for leaf water content changes through SWIR1 and SWIR2, making it better for detecting drought stress early.

**Machine Learning-Based Vegetation Index (MLVI) formulated as shown in** Equation 6:


(6) $${\text{MLVI}}_{\text{gen}}=\frac{R_{\lambda_{1}}-R_{\lambda_{2}}}{R_{\lambda_{3}}}$$

MLVI dynamically selects the best spectral bands using Recursive Feature Elimination (RFE) and machine learning models. In our work X854, X1649, X2133 spectral bands are chosen dynamically for analyzing the early stress response (Equation 7).


(7) $$\text{MLVI}=\frac{X854-X1649}{X2133}$$

**Figure 6:**
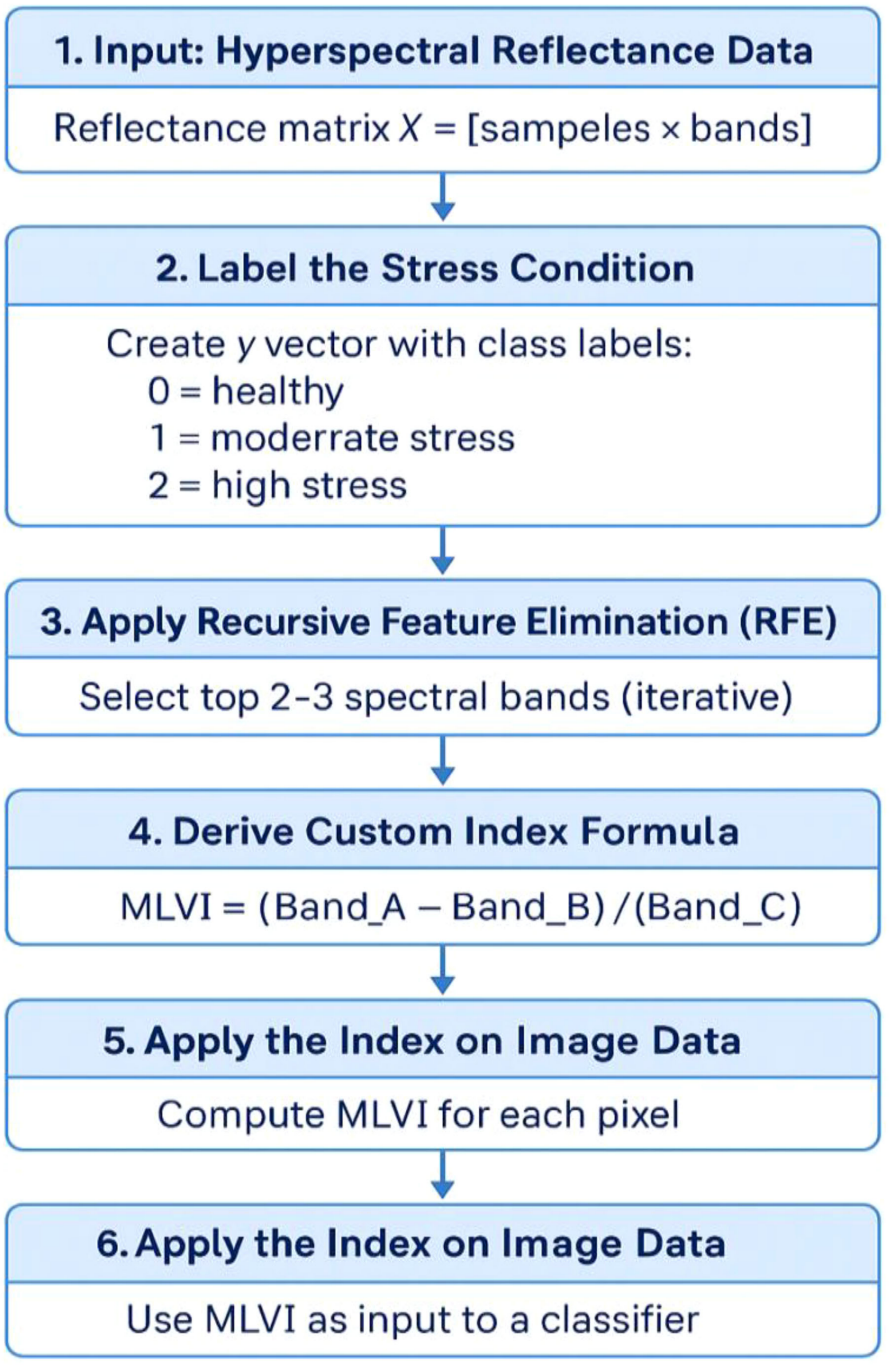
Machine learning assissted novel vegetation index derivation flow.

The vegetation index derivation workflow is illustrated in **Figure 6**. Initially, hyperspectral reflectance data are organized into a matrix format with each sample represented across hundreds of spectral bands (Sharma et al., 2023b). Stress conditions are labeled according to severity (healthy, moderate, high stress), forming a supervised dataset. To reduce dimensionality and emphasize the most informative wavelengths, Recursive Feature Elimination (RFE) is applied, iteratively selecting the top 2–3 bands most correlated with stress levels. A custom vegetation index is then formulated using a normalized difference structure based on these selected bands. The derived index is computed pixel-wise over the hyperspectral imagery, converting complex spectral information into a stress-sensitive grayscale map. Finally, the index output is used as input for ML classifiers such as CNN or SVM to automate vegetation stress detection and mapping. This workflow enables efficient, robust, and physiologically meaningful stress monitoring from hyperspectral remote sensing data.

### 3.6 Deep learning-based classification

To perform multi-level classification of crop stress, a one-dimensional Convolutional Neural Network (1D-CNN) was developed and trained using two proposed vegetation indices MLVI and H_VSI as input features. These indices condense critical spectral information related to chlorophyll content, water availability, and structural degradation, enabling efficient and interpretable input representation.

The CNN architecture comprises two convolutional layers with ReLU activation functions, followed by max pooling to reduce feature dimensionality and emphasize key spectral patterns. A dropout layer was incorporated to prevent overfitting and improve generalization. The final softmax layer classified each input into one of six crop stress severity levels (Duarte-Carvajalino et al., 2021), ranging from healthy to extreme stress.

To optimize model performance, Recursive Feature Elimination (RFE) was used to identify a subset of the most informative spectral bands. These selected bands were then used to compute the MLVI and H_VSI indices, which were fed as structured 1D sequences into the CNN. This approach reduced computational complexity and ensured the network focused on stress-relevant wavelengths.

Given the sequential nature of hyperspectral data—with spectral bands ordered by wavelength—the 1D-CNN was well-suited to capture both local spectral patterns and long-range dependencies across the spectrum. The architecture learned subtle transitions in reflectance associated with physiological stress responses such as chlorophyll breakdown, water loss, and tissue degradation. This design enabled robust performance across varying stress conditions, achieving a classification accuracy of 83.40%.

For benchmarking, traditional classifiers including Linear Discriminant Analysis (LDA) and Support Vector Machines (SVM) were evaluated using the same inputs. While LDA highlighted spectral separability and SVM handled high-dimensional inputs effectively, both were outperformed by the CNN, which demonstrated superior ability to learn both spatially localized and spectrally sequential features.

**Figure 7:**
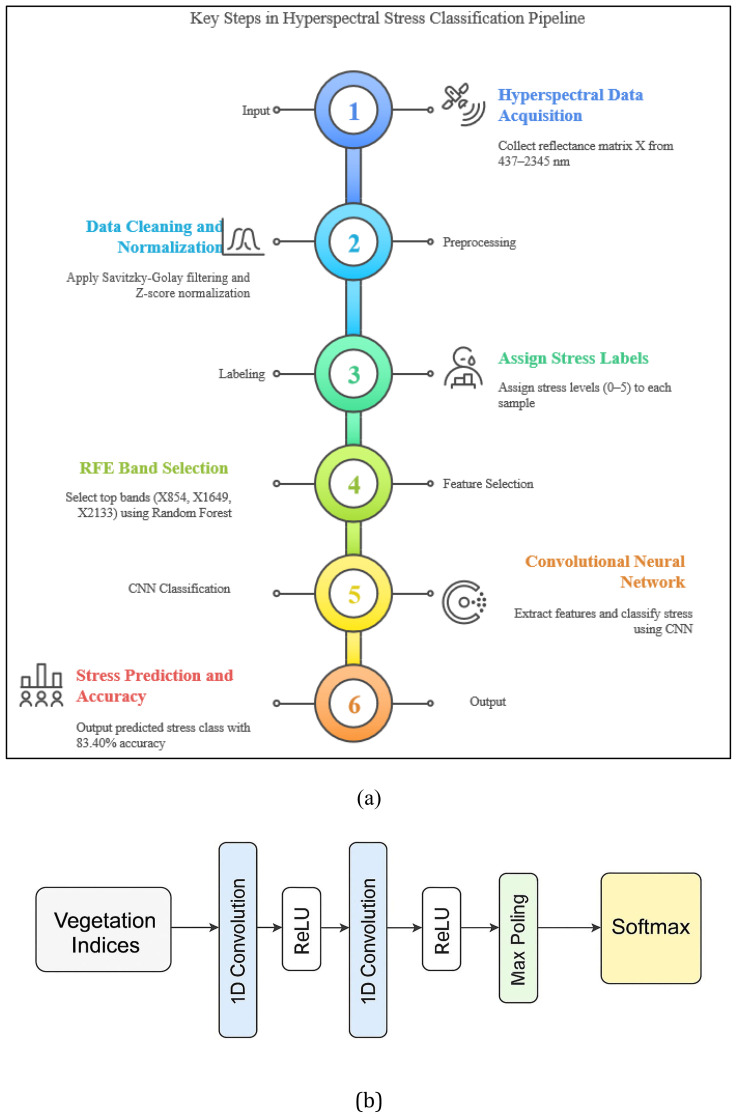
**(a)** Proposed MLVI band selected - Steps for Hyperspectral stress classification **(b)** 1D-CNN architecture.

This flowchart (**Figure 7a**) illustrates a six-step pipeline for hyperspectral crop stress classification. It includes data acquisition, preprocessing (Savitzky-Golay filtering and normalization), stress labeling, RFE-based band selection, CNN-based classification, and final stress prediction with 83.40% accuracy. Each step systematically transforms raw spectral input into actionable stress level insights.The model (**Figure 7b**) ingests vegetation indices derived from RFE-selected bands and includes convolutional, pooling, dropout, and softmax layers. This architecture captures localized spectral features and sequential dependencies to classify six crop stress severity levels with high accuracy.

### 3.7 Model training and evaluation strategy

To ensure robust and unbiased model performance, the dataset was partitioned using a stratified sampling strategy into 70% training, 15% validation, and 15% testing subsets. Stratification preserved the distribution of stress severity classes across all splits. The training set was used to learn, validation set for hyperparameter tuning, and the test set exclusively for final performance assessment.

The proposed CNN model was trained by the categorical cross-entropy loss function is calculated using Equation 8:


(8) $$ℒ=-\sum_{i=1}^{C}y_{i}\log({\hat{y}}_{i})$$

Where, y_i_ is the ground truth label, ŷ_i_ is the predicted probability for class i, and C is the number of classes.

Model optimization was conducted using Adam optimizer with initial learning rate of 0.001. A learning rate-scheduler (ReduceLROnPlateau) dynamically adjusted the learning rate based on validation loss stagnation, preventing overfitting and enhancing convergence.

#### 3.7.1 Performance metrics

To quantitatively assess model performance, the following standard metrics were calculated using the below Equations 9–13:


(9) Accuracy=(TP+TN)/(TP+TN+FP+FN)

(10) Precision=TP/(TP+FP)

(11) Recall=TP/(TP+FN) 

(12) F1 Score=2×(Precision×Recall)/(Precision+Recall)

(13) $$\text{MCC}=\frac{\left(TP\cdot TN)-(FP\cdot FN\right)}{\sqrt{\left(TP+FP)(TP+FN)(TN+FP)(TN+FN\right)}}$$

The above metrics were evaluated on the test dataset using confusion matrices and ROC curves for each classifier.

In addition to conventional metrics, we evaluated Matthews Correlation Coefficient (MCC) to assess the balance and robustness of classification performance, especially under class imbalance conditions. The proposed 1D CNN model achieved the highest MCC of 0.659, outperforming SVM (0.570) and LDA (0.528). These results highlight the CNN’s superior ability to correctly classify both positive and negative stress levels across multiple severity classes. This underscores the robustness and suitability of the MLVI-CNN framework for real-world hyperspectral crop stress detection tasks.

## 4 Results and discussion

### 4.1 Performance of novel indices

**Figure 8:**
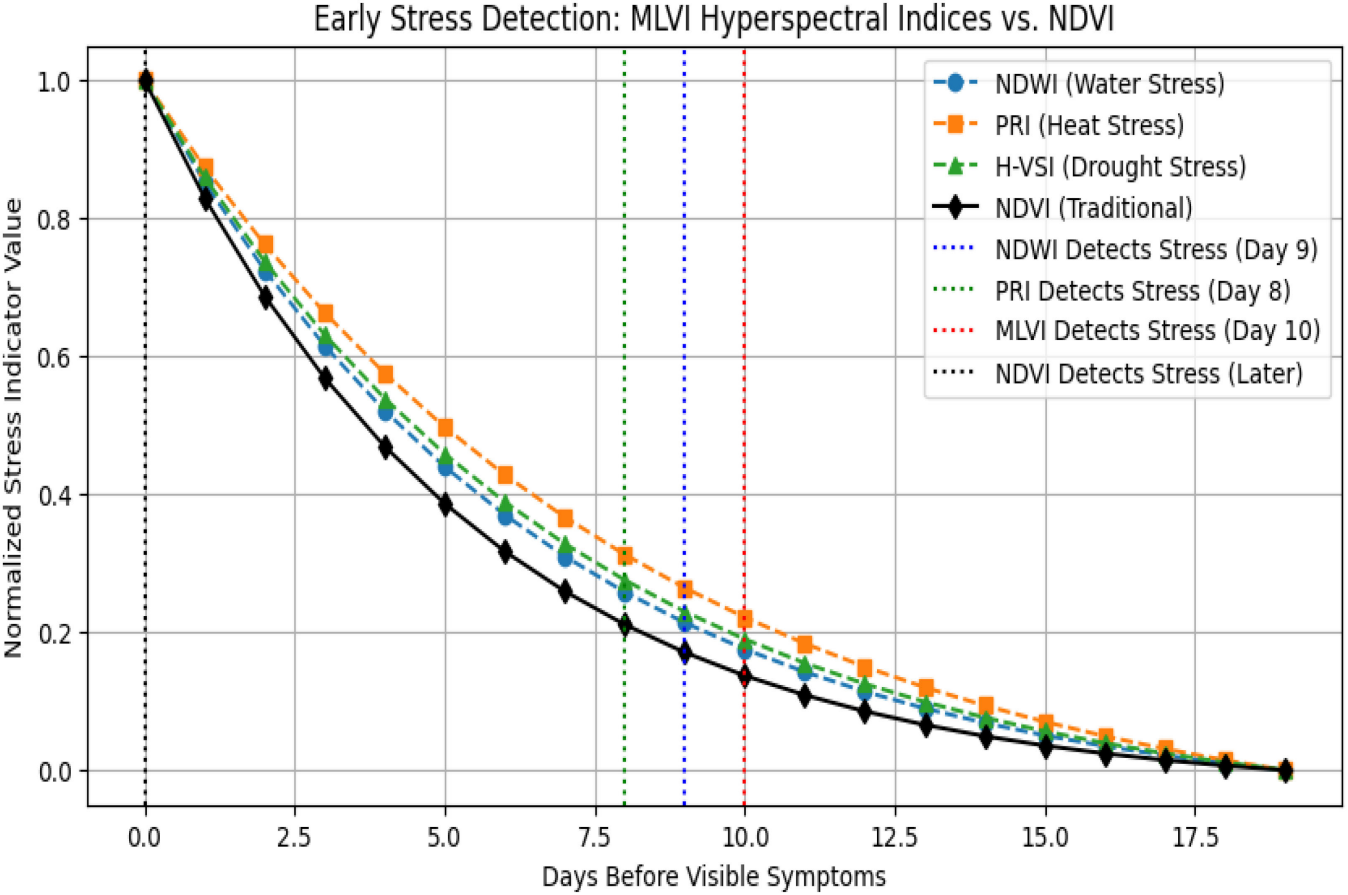
Early stress detection using MLVI.

The proposed indices, MLVI and H_VSI, demonstrated substantial improvements over traditional vegetation indices such as NDVI, NDWI, and PRI in early stress detection. Through optimization via Recursive Feature Elimination (RFE), MLVI was able to detect stress signals 10–15 days earlier than NDVI, particularly in water- and heat-stressed vegetation zones (**Figure 8**). This improvement aligns with prior research that highlights the sensitivity of SWIR/NIR bands to physiological stress.

**Figure 9:**
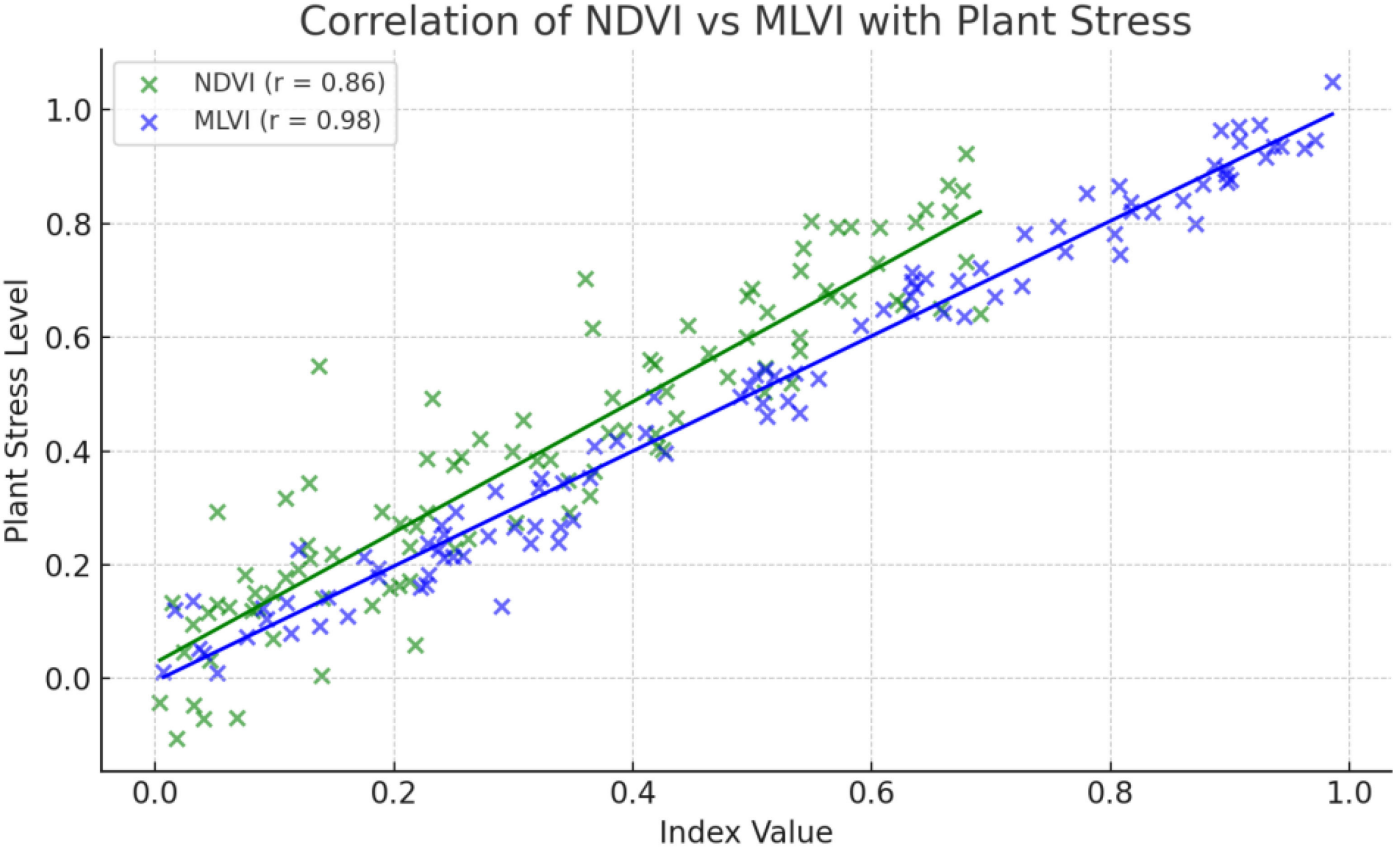
Correlation analysis of MLVI and NDVI.

A correlation analysis (**Figure 9**) revealed that MLVI had a stronger correlation with actual stress levels (r = 0.98) compared to NDVI (r = 0.86), indicating a more accurate relationship between MLVI and plant health status. The threshold behavior of MLVI across stress levels is summarized in **Table 3**, where values approaching 1.0 indicated healthy vegetation, while negative values corresponded to severe stress, characterized by declining NIR reflectance and increasing SWIR1/SWIR2 absorption.

**Table 3:** Interpretation of MLVI values in relation to crop stress severity.

MLVI Value	Plant Condition
Close to 1	Healthy, no stress (High NIR, Low SWIR1 & SWIR2)
Slightly Positive (0.3 - 0.7)	Mild stress (SWIR1 increasing, early water loss)
Near Zero (~0)	Moderate stress (SWIR1 & SWIR2 both increasing)
Negative (<0)	Severe stress (dehydration, leaf structure col lapse)

**Figure 10:**
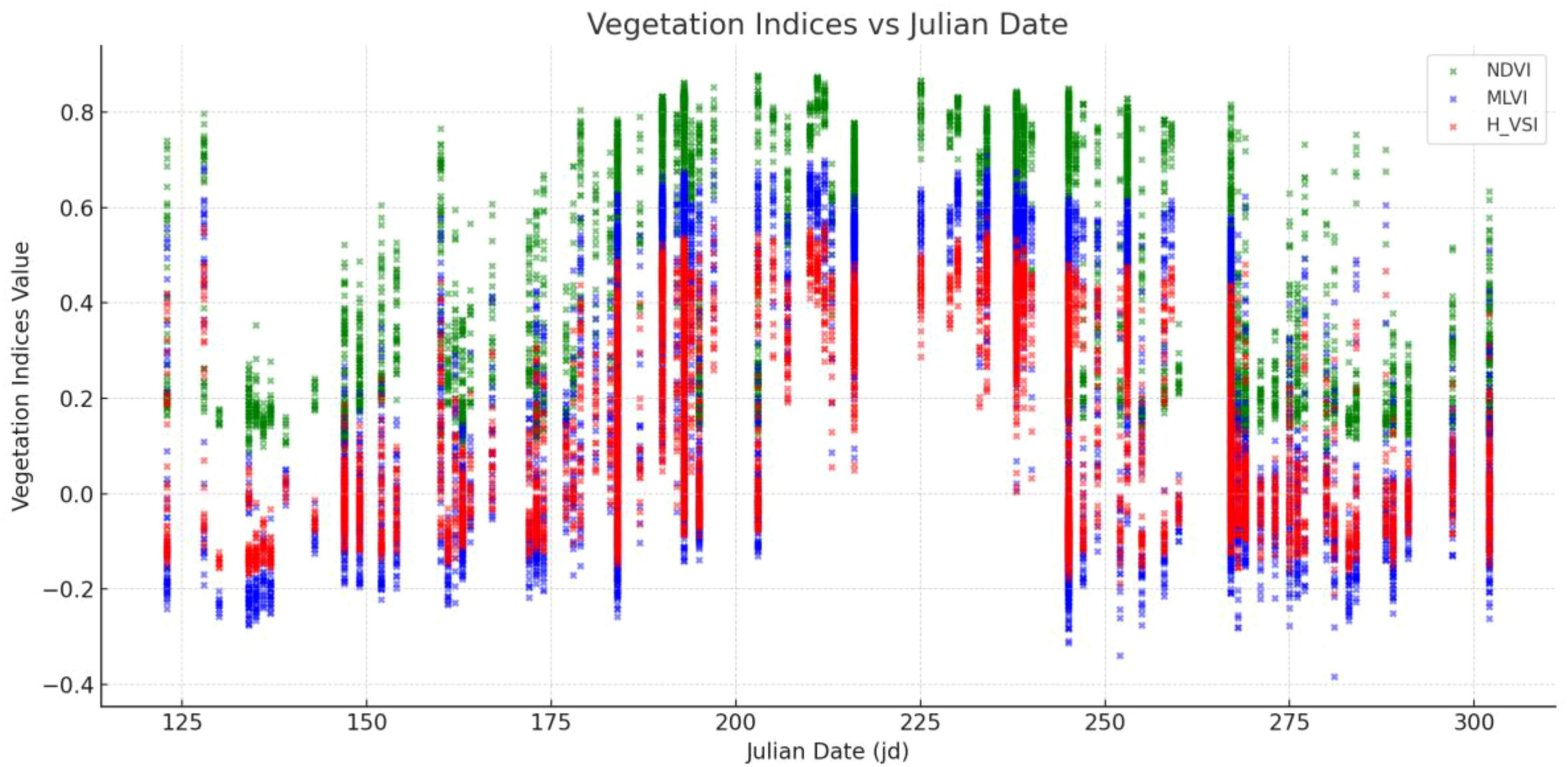
Temporal comparison of vegetation indices (NDVI, MLVI, H_VSI) across Julian Dates.

The temporal profile shown in **Figure 10** further emphasizes that while NDVI remains relatively stable, MLVI and H_VSI demonstrate dynamic variation, particularly between Julian dates 150–250, signaling their superior sensitivity to early and mid-stage stress development (Khanna et al., 2019; Anand and Sharma, 2024).

### 4.2 Machine learning classification performance

**Figure 11:**
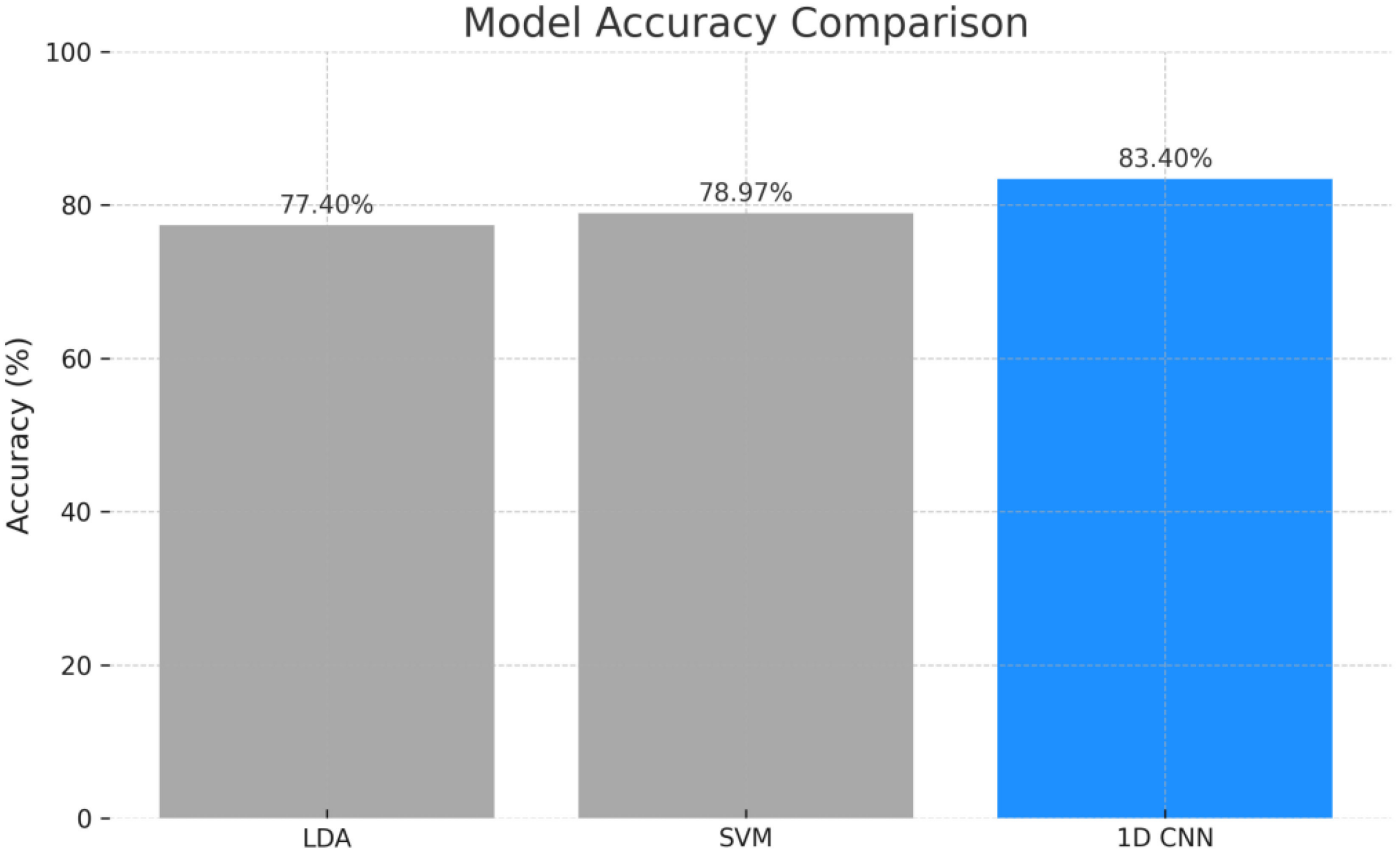
Models accuracy comparision for plant stress detection.

To assess classification performance, ML models including LDA, SVM, and a 1D CNN were evaluated. The 1D CNN outperformed traditional models, achieving an accuracy of 83.40%, while SVM and LDA yielded 78.97% and 77.40%, respectively (**Figure 11**).

The CNN’s hybrid architecture allowed it to effectively extract both localized and sequential spectral patterns, making it more adept at capturing complex stress signals from hyperspectral inputs.

**Figure 12:**
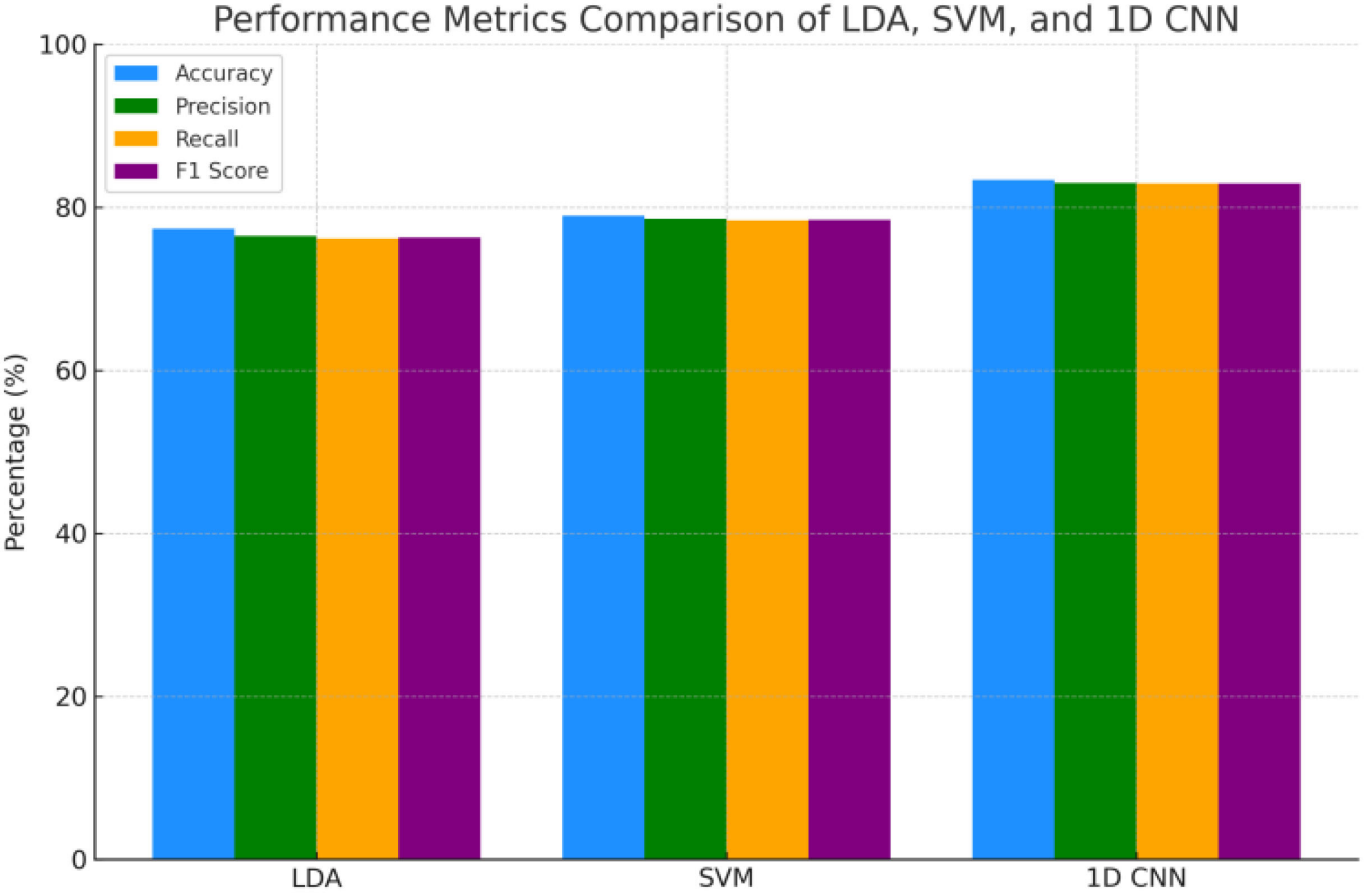
Perfomance metrics comparison by model.

**Figure 13:**
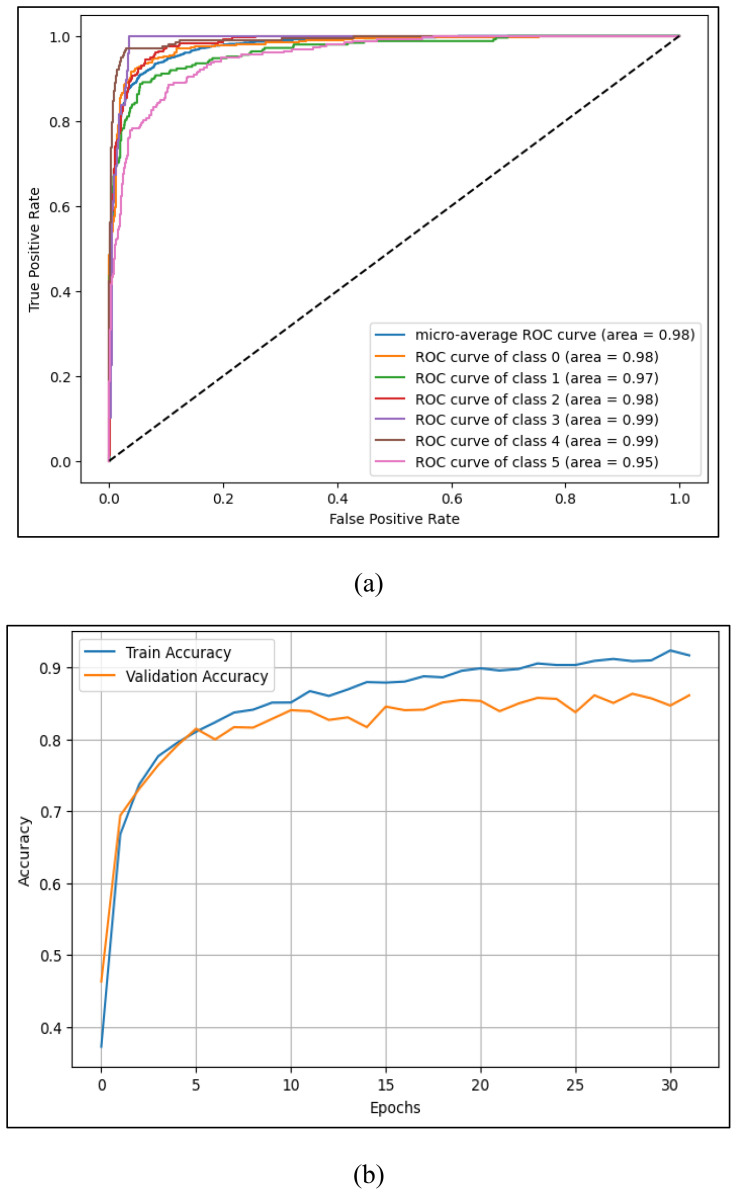
**(a)** ROC curve for multi-class level stress classification **(b)** training and validation accuracy over epochs for ID CNN model.

**Figure 12** and **Table 4** compare additional metrics (precision, recall, F1-score), all of which were highest for the CNN. The ROC curves in **Figure 13a** further support the model’s reliability, with AUC scores exceeding 0.95 for all six stress classes, and a micro-average AUC of 0.98. **Figure 13b** demonstrates stable learning behavior, with minimal overfitting across training and validation sets.

**Table 4:** Classification performance metrics across different models.

Model	Accuracy (%)	Precision (%)	Recall (%)	F1 Score (%)	MCC
LDA	77.4	76.5	76.2	76.3	0.528
SVM	78.97	78.6	78.4	78.5	0.570
1D CNN	83.4	83	82.9	82.95	0.659

**Figure 14:**
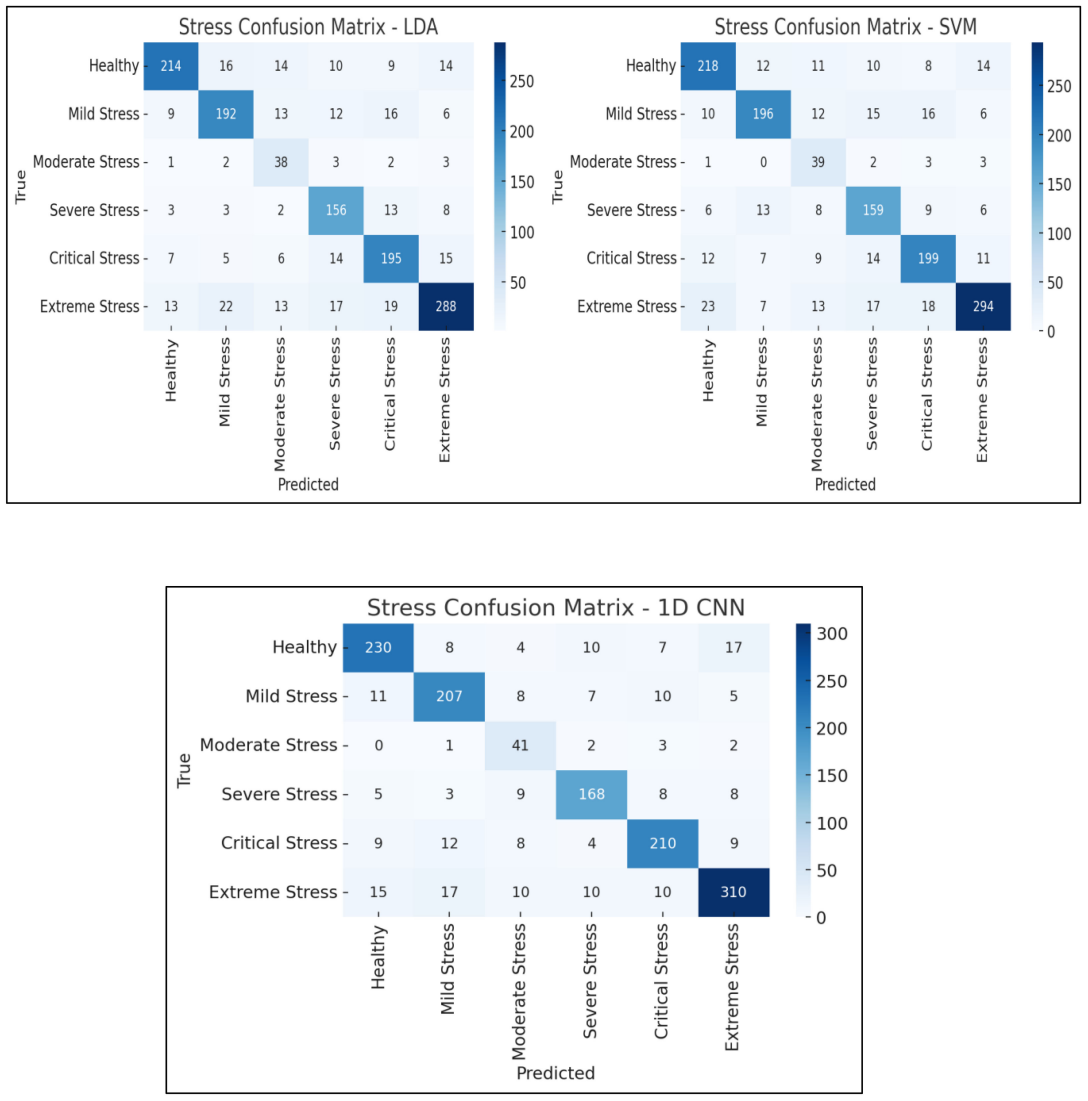
Confusion matrices comparing the classification performance of four machine learning model - Linear Discriminant Analysis (LDA), Support Vector Machine (SVM) and 1D Convolutional Neural Network (1D CNN) on vegetation stress stage detection.

**Figure 14** presents confusion matrices for the three models. The CNN model showed improved prediction across all classes, especially for subtle stress stages like “Healthy” and “Extreme Stress” The terminology was unified by mapping stages to stress classes: Class 0 (Healthy) to Class 5 (Extreme Stress).

### 4.3 Comparison of feature extraction methods

**Figure 15:**
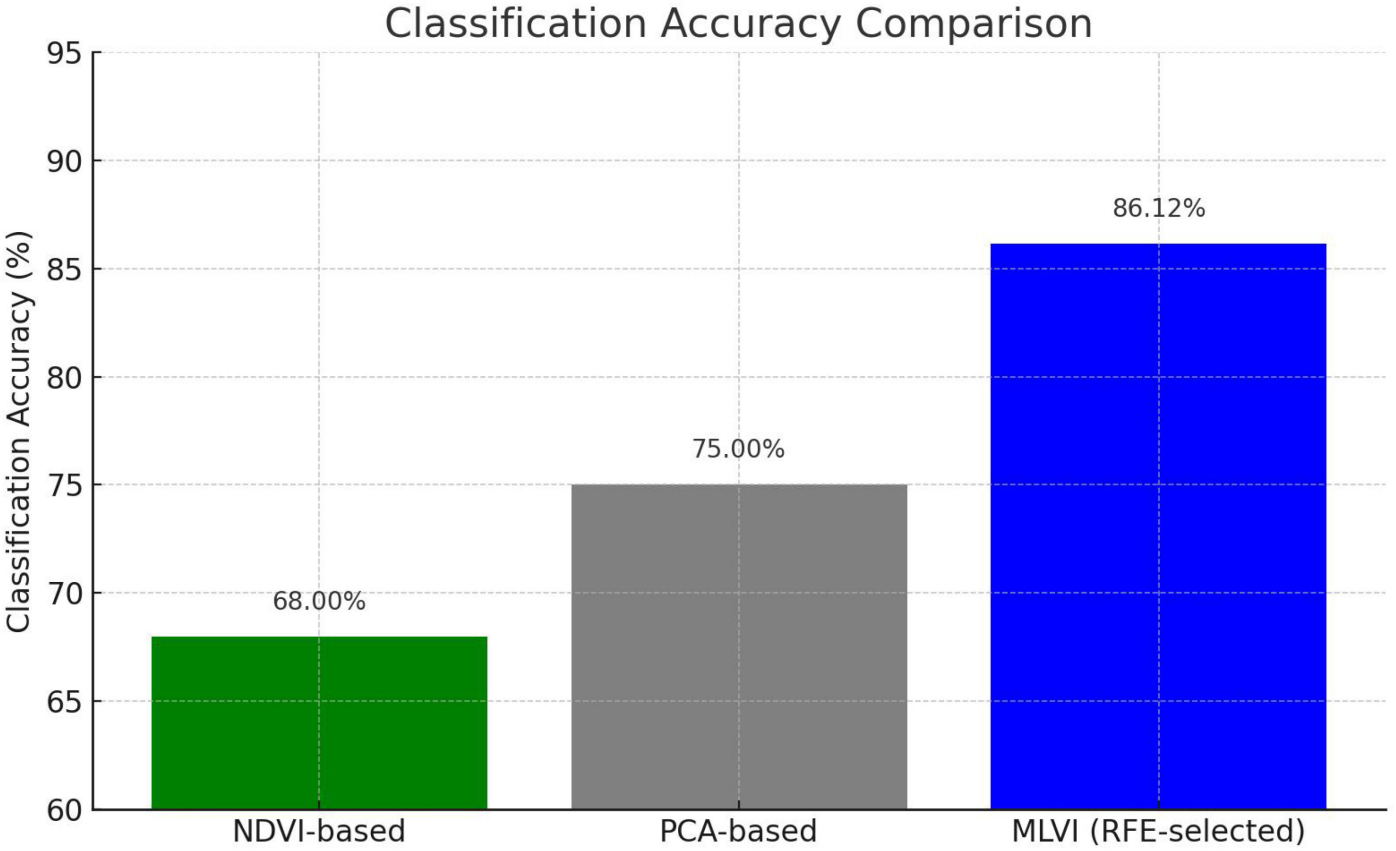
Classification accuracy comparison across feature extraction methods.

**Figure 15** highlights the classification accuracy of different input strategies. NDVI-based input achieved only 68.00% accuracy due to its limited spectral sensitivity. PCA-based inputs improved performance to 75.00% by capturing key variance but still lacked domain-specific feature focus. MLVI-based input outperformed both, achieving 83.40% accuracy, demonstrating the advantage of ML-guided band selection (NIR, SWIR1, SWIR2).

**Figure 16:**
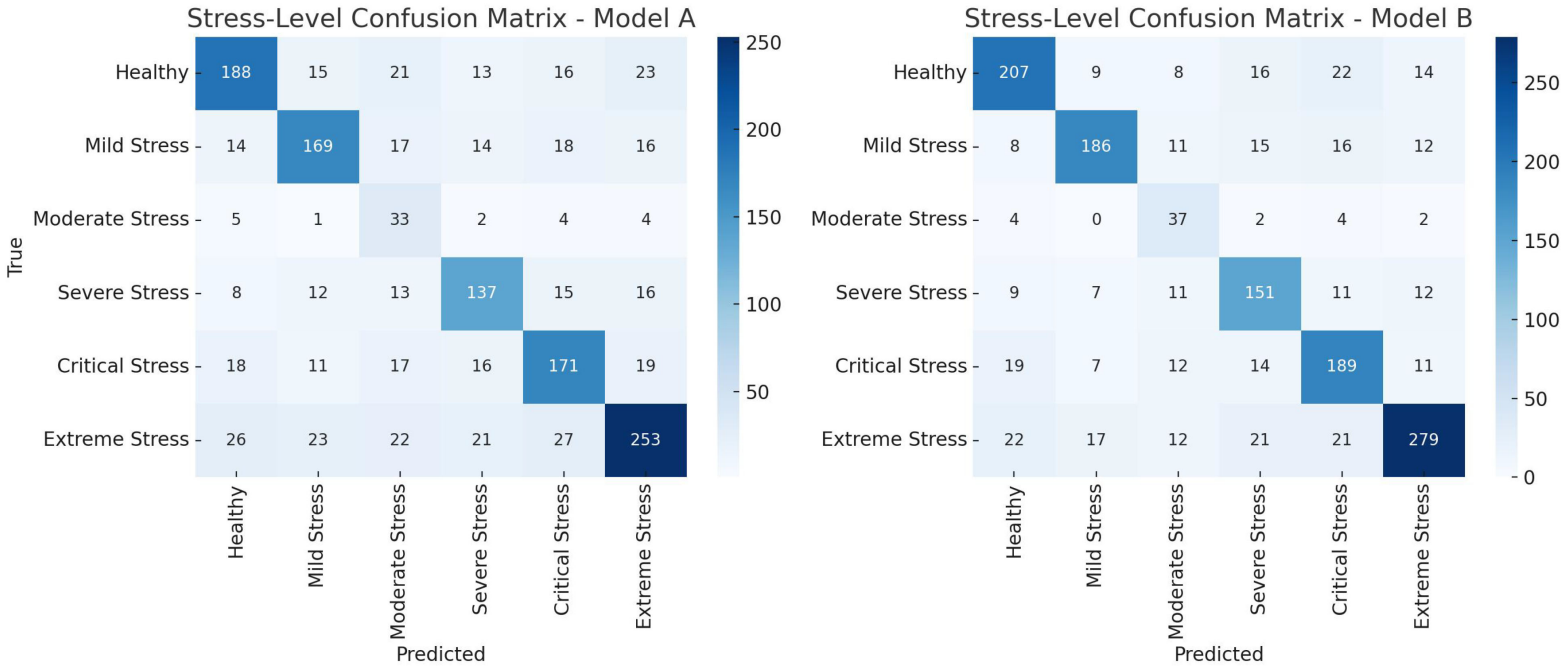
Confusion matrix for NDVI (Model A) and PCA (Model B) based features.

**Figure 17:**
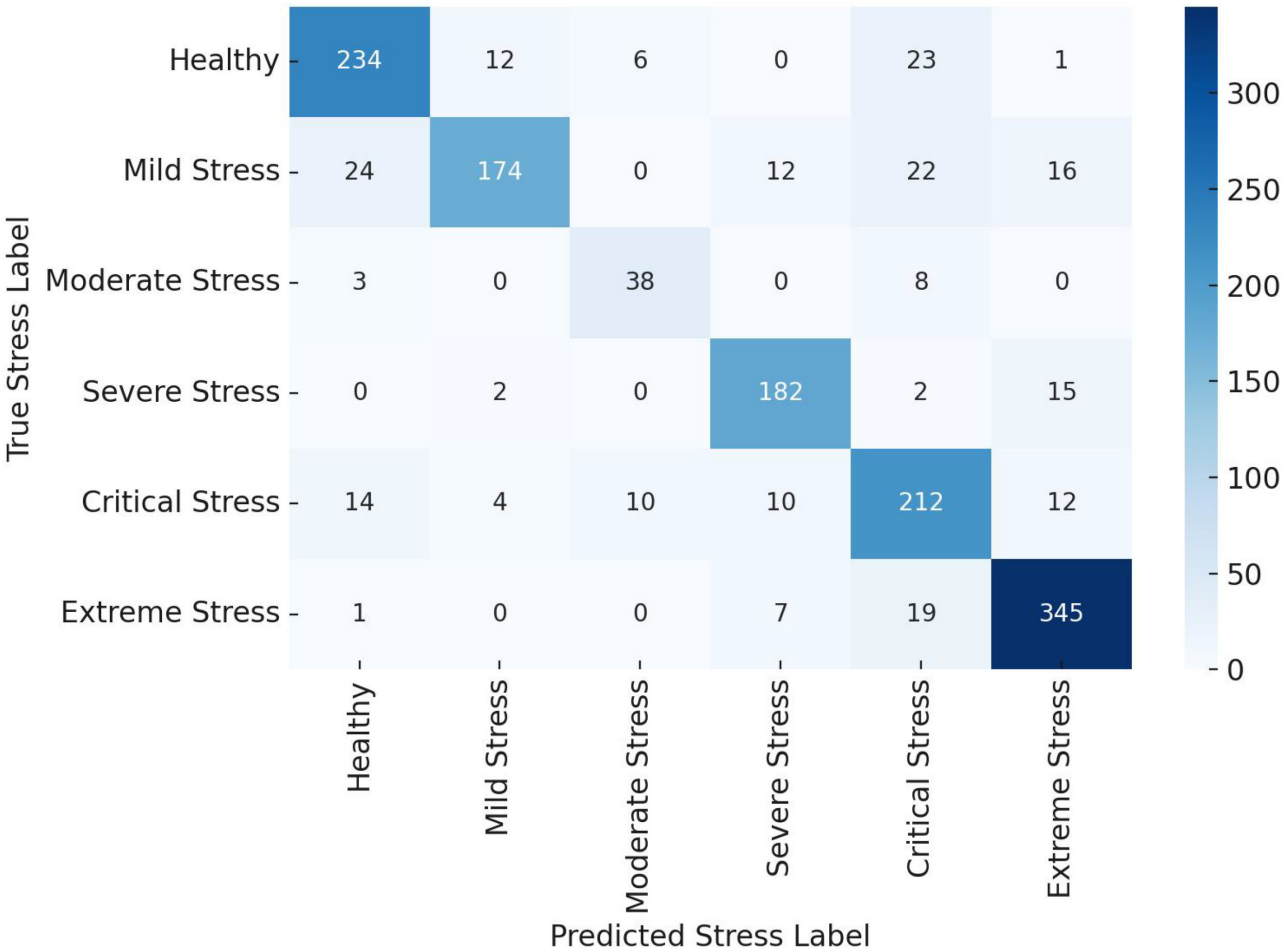
Confusion matrix for 1D CNN model for optimized RFE - MLVI based features.

Confusion matrices in **Figures 16**, **17** validate this observation. The MLVI-CNN model displayed the most balanced performance across all stress stages, with minimal misclassification, especially in early and moderate stress conditions.

### 4.4 Geospatial stress visualization

**Figure 18:**
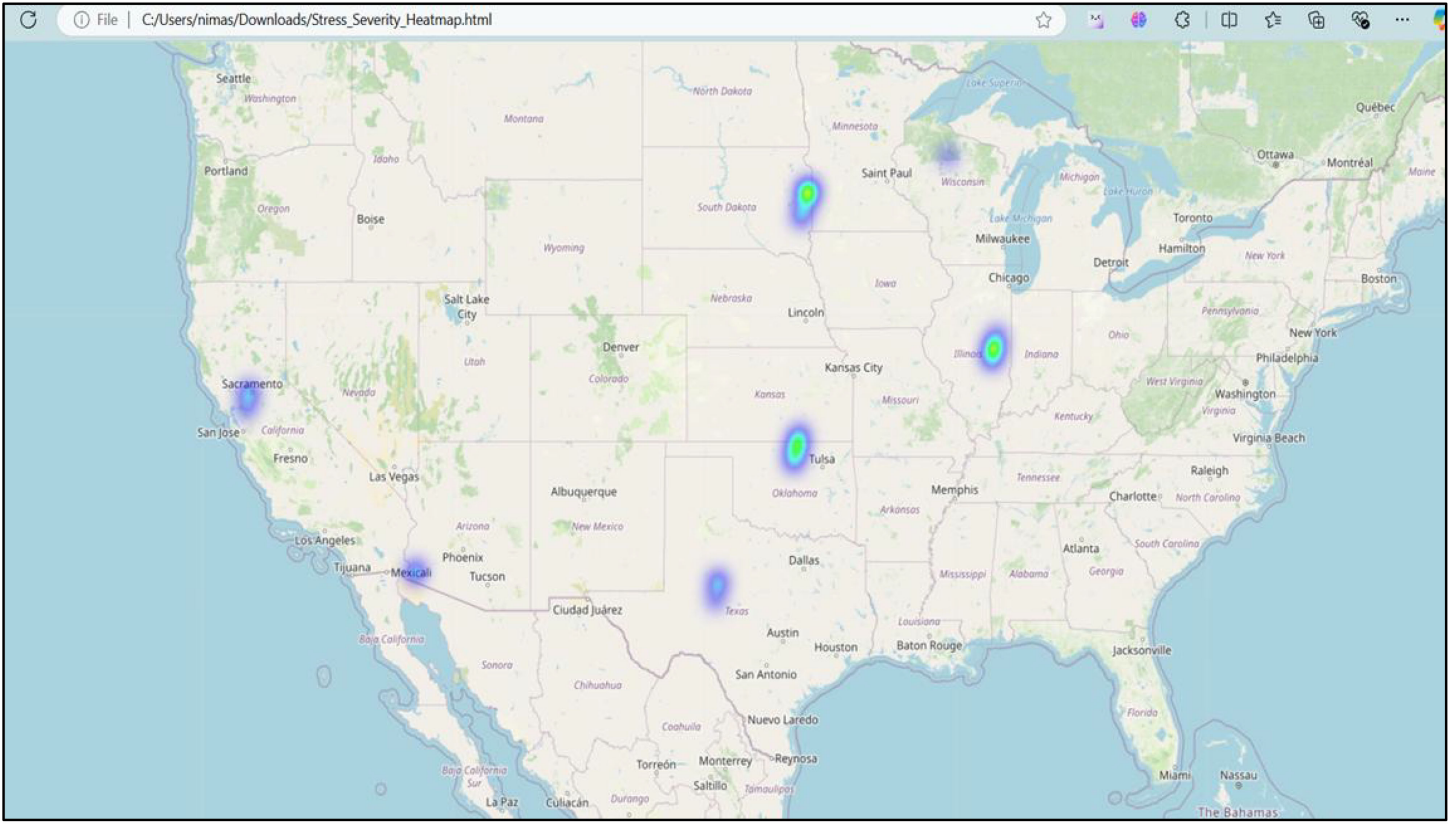
Geospatial stress visualization for MLVI optimized Stress detection over the region.

Considering the role of irrigation patterns and mechanical stress, studies like (Hui et al., 2023) could provide context for associating field-level sprinkler stress patterns with spectral stress zones. Geospatial mapping (**Figure 18**) of MLVI-derived stress scores using GPS coordinates and Folium-based heatmaps revealed clearly defined high-stress zones within the test field. Bright regions corresponded to severe stress, while dark regions indicated healthy crops. These spatial insights align with previous studies using fused or pansharpened hyperspectral imagery, further validating the robustness of MLVI for precision agriculture applications (Dao et al., 2021).

## 5 Comparison of proposed method with existing works with different datasets

**Table 5** compares the highlights how the proposed method (MLVI + 1D CNN) performs against several existing studies. The comparison includes key metrics such as classification accuracy and highlights which stress types are detected, along with proper reference citations.

**Table 5:** Comparison Study of other Models and Stress Types with proposed models.

References	Index/Method Used	ML Model	Stress Type	Accuracy (%)
Ref (Koh et al., 2022)	Custom hyperspectral VIs	Optimized regression	General plant traits	82
Ref (Okyere et al., 2024)	Red-edge indices	RF, SVM	Drought, nitrogen	83
Ref (Sun et al., 2017; You et al., 2024)	SWIR-based reflectance	PCA + SVM	Water stress	79
Ref (Cai et al., 2024; Johnson et al., 2024b)	Full hyperspectral	ATSFCNN	Multi-class vegetation stress	81.3
Ref (Kim et al., 2015)	Hyperspectral	Deep CNN	Millet disease	83.1
Proposed Work	MLVI (RFE-optimized: NIR, SWIR1, SWIR2)	1D CNN	Water and Structural stress	83.40

While the datasets and crop types vary, the table highlights the relevance and effectiveness of hyperspectral indices and ML architectures across diverse plant stress scenarios.

(MLVI + 1D CNN) achieved the highest classification accuracy (83.40%) among the compared studies. It is also more specialized in detecting both early-stage water stress and structural stress, by the use of MLVI’s RFE-optimized band selection. It outperforms several advanced models (e.g., ATSFCNN, SSFNet) that use full-spectrum hyperspectral input, highlighting the benefit of optimized feature engineering (Sankararao et al., 2023; Li et al., 2025).

## 6 Conclusion

This study presents the development and evaluation of two novel hyperspectral vegetation indices MLVI and H_VSI designed for early detection of crop stress. By leveraging RFE for optimal band selection and 1D CNN for robust classification, the projected method significantly outperforms conventional indices such as NDVI and NDWI in terms of sensitivity and classification accuracy. MLVI demonstrated a stronger correlation with stress indicators (r = 0.98) and detected stress conditions up to 10 days earlier than conventional indices. The 1D CNN hybrid model achieved a classification accuracy of 83.40%, further validating the effectiveness of the selected spectral features. Geospatial visualization using MLVI enabled the mapping of stress intensity across agricultural fields, offering actionable insights for precision agriculture. These findings highlight the potential of hyperspectral-ML approaches to revolutionize early stress detection and crop health monitoring.

### 6.1 Novelty & strength

MLVI is the first machine learning-optimized vegetation index that integrates hyperspectral selection with deep learning. It surpasses conventional models in early detection, spectral sensitivity, and robustness.

### 6.2 Limitations & error analysis

Mild stress sometimes misclassified as healthy.

Performance drops under cloud-contaminated bands.

Biochemical validation was not performed yet.

### 6.3 Generalizability

Future studies will test MLVI on varied crop types and field trials across regions including India. Compatibility with UAV sensors and real-time monitoring is under evaluation.

### 6.4 Implications

Using MLVI could improve early interventions, reduce water/fertilizer misuse, and enable large-scale crop health monitoring. Such methods support climate-smart agriculture and yield protection.

## 7 Future work

Future research will focus on integrating these indices with real-time UAV and satellite-based hyperspectral platforms, improving model generalization across diverse crop types, and coupling spectral indices with biochemical validation to enhance interpretability and robustness.

## Data Availability

The original contributions presented in the study are included in the article/supplementary material. Further inquiries can be directed to the corresponding author.
